# Ash production by attrition in volcanic conduits and plumes

**DOI:** 10.1038/s41598-017-05450-6

**Published:** 2017-07-17

**Authors:** T. J. Jones, J. K. Russell

**Affiliations:** 1 Department of Earth Sciences, Durham University, South Road, Durham, DH1 3LE UK; 2 Department of Earth, Ocean & Atmospheric Sciences, University of British Columbia, Vancouver, British Columbia V6T 1Z4 Canada

## Abstract

Tephra deposits result from explosive volcanic eruption and serve as indirect probes into fragmentation processes operating in subsurface volcanic conduits. Primary magmatic fragmentation creates a population of pyroclasts through volatile-driven decompression during conduit ascent. In this study, we explore the role that secondary fragmentation, specifically attrition, has in transforming primary pyroclasts upon transport in volcanic conduits and plumes. We utilize total grain size distributions from a suite of natural and experimentally produced tephra to show that attrition is likely to occur in all explosive volcanic eruptions. Our experimental results indicate that fine ash production and surface area generation is fast (<15 min) thereby rapidly raising the fractal dimension of tephra deposits. Furthermore, a new metric, the Entropy of Information, is introduced to quantify the degree of attrition (secondary fragmentation) from grain size data. Attrition elevates fine ash production which, in turn, has consequences for eruption column stability, tephra dispersal, aggregation, volcanic lightening generation, and has concomitant effects on aviation safety and Earth’s climate.

## Introduction

Ash released by explosive volcanic eruption can adversely affect air traffic^1, 2^, human health^3^, agriculture^4^, urban infrastructure and contributes to short term global climate change^5^. Eruptions featuring higher fine ash contents pose greater risk. Despite these significant societal impacts, the timing, rates, and sources of fine ash generation within explosive eruptions remain controversial. Total grain size distributions (TGSDs) of pyroclastic deposits indirectly inform on volcanic fragmentation processes. However, many TGSDs are bimodal suggesting multiple mechanisms, or events, of fragmentation and for which there is no single explanation^6^. Here, we show that attrition, driven by particle-particle interactions, is an efficient form of secondary fragmentation that must operate in all explosive eruptions. Experiments on pumice provide rates of ash production and support a preferential grain size reduction with increasing fragmentation depth and eruption column height. Attrition-driven ash production impacts eruption column stability, tephra dispersal, aggregation, volcanic lightening generation, and has concomitant effects on aviation safety and Earth’s climate.

**Figure 1:**
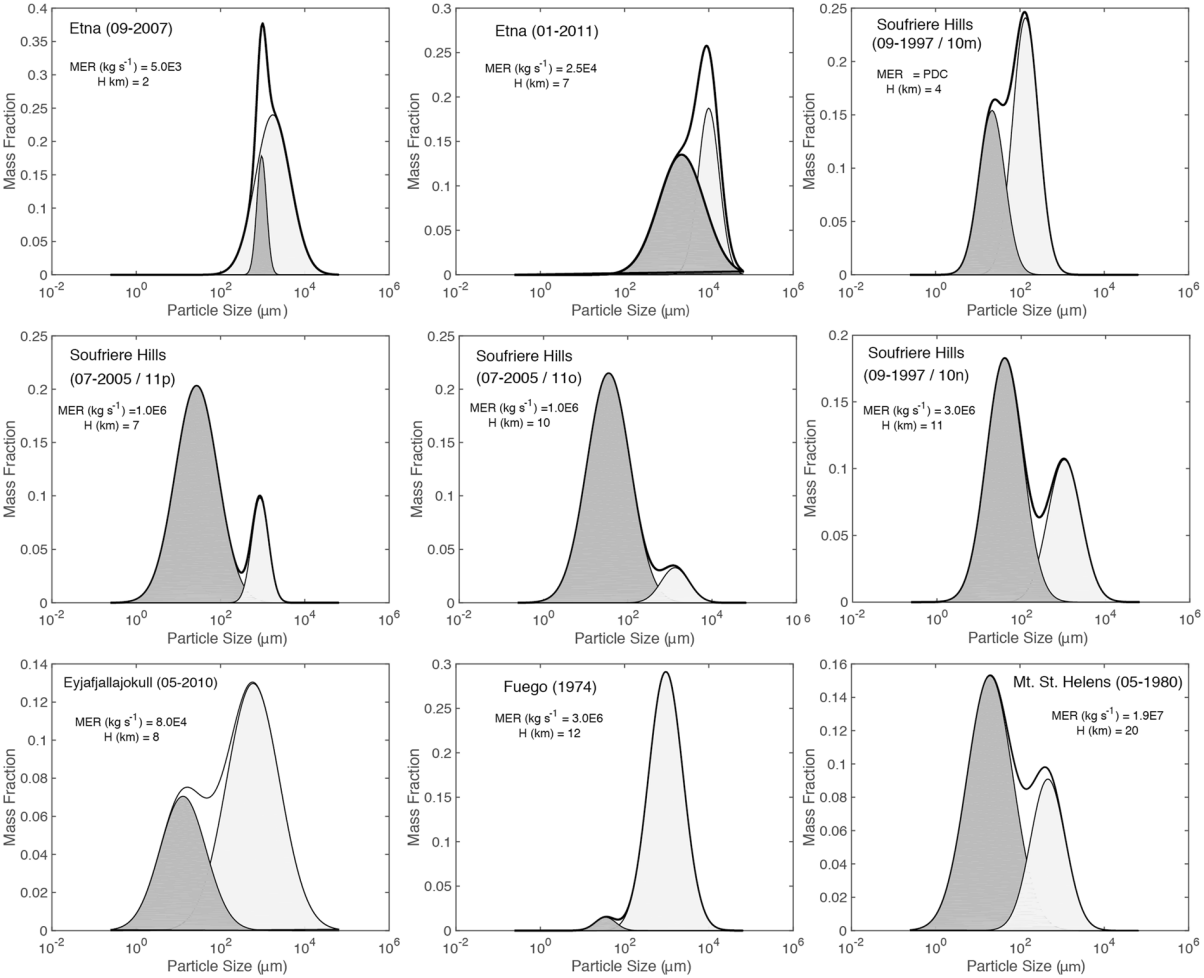
Total grain size distribution curves for tephra deposits from a variety of modern explosive volcanic eruptions as compiled and modelled by Costa *et al*. (2016)^6^. They are re-plotted here as particle size (μm) *versus* mass fraction. The original TGSD curve from Costa *et al*. (2016)^6^ is represented by a heavy black line and the two model distributions fitted to the TGSD curve in this study are shown as light (coarse) and dark (fines) fields. The corresponding mass eruption rate (MER) and plume height (H) area shown within each sub-panel.

Tephra deposits result from explosive volcanic eruption and serve as indirect probes into fragmentation processes operating in subsurface volcanic conduits. Most ash results from magmatic fragmentation within the subterranean conduit wherein volatile exsolution and expansion transform the rising bubbly magma into a gas jet loaded with a chaotic suspension of poorly sorted fragments of the disrupted magma^7^. In principle, the reconstructed TGSD’s and componentry of the tephra deposits indirectly inform on the depths and mechanisms of fragmentation, the state of the magma at the fragmentation surface, and the total energy released by explosive fragmentation^8, 9^. Additionally, TGSD’s are used as a proxy for the initial size distributions of tephra produced by primary fragmentation; a vital source parameter in ash dispersion models^10^. However, this assumes tephra production derives from a single primary fragmentation mechanism and that particles are not subjected to secondary fragmentation processes^11–15^. Close examination of TGSD’s for a wide range of eruptions shows that most are not simple unimodal normal distributions (Fig. 1). Rather, the TGSDs are best modelled as bimodal distributions and such bimodality has been previously ascribed to inputs from pyroclastic density currents^10, 16^ (by secondary fragmentation), magma heterogeneity^6^, or to contributions from a phreatomagmatic component^6^. The degree of bimodality varies (Fig. 1). Some eruptions show two, unique, well-defined modes such as the 1997 Soufriere Hills eruption whilst others, like products from the 2007 Etna eruption, are less clearly defined.

Secondary fragmentation is defined as the reduction in particle grain size supervening primary magmatic fragmentation and can involve a wide variety of mechanisms. Here we focus on particle size reduction by attrition^17, 18^. Attrition can occur by particle-particle disruptive collisions^12, 18^ where the impact energy is sufficiently high to break particles into a series of daughter fragments^18^. Attrition also includes abrasion^18^, which involves surface chipping of irregularities on the exterior surfaces of particles. Secondary fragmentation has, for example, been extensively linked to transport within pyroclastic density currents (PDC’s)^16, 19^. Within PDC’s secondary ash formation dominantly occurs through abrasion leading to a rounded particle morphology^11, 20, 21^. Controlled laboratory investigations and numerical models have shown that ash production increases with transport distance but is most effective at vent proximal locations where energy-levels are highest^15, 22, 23^. Abrasion and milling within PDC’s round pumice clasts^21^, strip vesicular material from crystals^11, 24^ and can have increased efficiency in the presence of lithics^25^. Secondary fragmentation can also operate within the subsurface conduit as the fragmented magma travels from the fragmentation front to the surface^11, 12, 14, 26, 27^. Dufek *et al*.^12^ showed that the break up of particles in the conduit is most successful when particles are >1 cm in diameter and fragmentation is deep. Furthermore, secondary fragmentation modifies the grain size characteristics, componentry and textures of tephra produced by magmatic fragmentation^11, 12^. However, particle concentrations within the eruption plume have been previously thought to be too low for particle attrition^14^.

Here, we present a model for fine ash production via secondary fragmentation within volcanic conduits and plumes. We suggest that attrition processes supported by particle-particle interactions constitute a pervasive form of secondary fragmentation that operates in all explosive eruptions. As such, fine ash production by attrition provides a simple explanation for the bimodal TGSD’s that characterize most tephra deposits. The concepts we present here are supported by a new suite of analogue experiments^18^ that use a standardized apparatus (ASTM 5757-00) to quantify attrition of pumice particles as a function of residence time in a jet (see methods)^18^. The experiments consist of an attrition tube wherein pumice particles of a known size distribution are entrained and suspended by a 10 L min^−1^ gas feed for a given time. A wider settling chamber is situated above the attrition tube causing a decrease in gas velocity and, thereby, allowing particles on the order of 10 μm in diameter (depending on local gas velocity and particle density) to settle and re-enter the attrition tube. Smaller ultra-fine particles have low Stokes settling velocities (i.e. less than gas velocity) and continue to rise and be collected in a fines bin. These ultra-fine particles are therefore unable to partake in further particle-particle interactions; however we expect such ultra-fine particles to contribute a negligible amount to attrition since their velocities are extremely low (≪0.0175 ms^−1^)^18^. Furthermore, particle segregation^28–30^, aggregation^31, 32^ and gravitational instabilities^33^ are known to operate in volcanic plumes providing simple and natural mechanisms for removing fines from, or creating spatial heterogeneities in, the plume.

Our analysis in this study utilizes the data collected from attrition experiments which constrain the rates, mechanisms and efficiency of pumice attrition in jets. These results, combined with quantitative TGSD descriptions from a suite of natural eruptions allow us to formulate an attrition model for volcanic eruptions.

## Results

### Attrition Experiments

**Figure 2:**
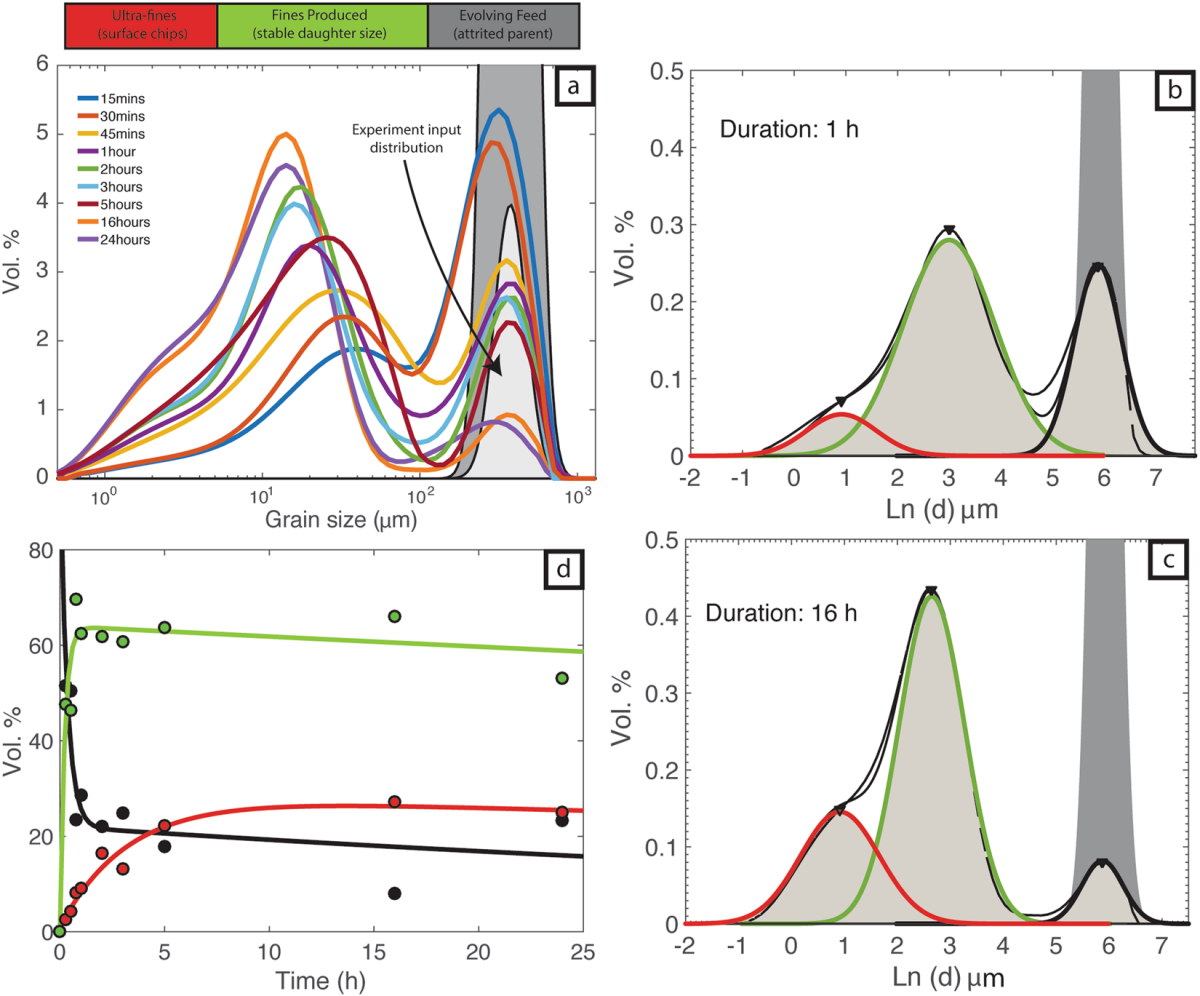
Grain size distributions as volume % for pumice in the jet attrition experiments. (**a**) Grain size distribution curves colour-coded for duration (15 min to 24 h) of attrition experiments. Modified from Jones *et al*.^18^. (**b**,**c**) Model normalized log normal distributions fitted to data derived from the 1 h and 16 h experiment respectively. The datasets are fitted to 3 normal (Gaussian) distributions whose medians, peaks, and standard deviations correspond to the parent particles, the daughter particles, and the ultrafine particles. The time zero model is calculated assuming a 375 μm median feed size and the variance is estimated assuming that the catching sieve sizes coincide with 3 s.d. (**d**) Summary of experimental results showing the changes in proportions (vol. %) of parent (black), daughter (green) and ultra-fine (red) particles.

The starting material for each experiment is a feed of pumice particles that has been sieved to a narrow size range (Fig. 2). Specifically, the pumice particles are nominally 250 μm collected between 250 and 500 μm mesh sieves. Each experiment subjects the initial pumice particles to attrition within the fixed gas jet for a controlled amount of time (i.e. a prescribed residence time). At the end of each experiment, the entire run-product is collected and processed for its TGSD (vol. % *vs*. particle size), which provides the data required to explore the relationship between grain size evolution and particle residence time (Fig. 2a). We note that, although some of the experimental durations are long compared to those experienced during natural transport, the experimental particle concentrations and velocities are several orders of magnitude lower than those observed in natural eruptions – suggesting that the experiments are inefficient relative to nature and provide minimum estimates of attrition rates. This will be further analysed in the discussion.

With increased duration the attrition experiments convert an initially unimodal TGSD of the feed particles into bimodal distributions (trimodal including the original feed). The particle attrition is driven by disruptive collisions^34^ that fragment parent particles into smaller-sized daughter products and by abrasive chipping of particle surfaces. Thus, the experimental products feature three distinctive grain size populations (Fig. 2a): (1) a feed of original or parent particles (500 to 250 μm) that become reduced in size and abundance by attrition (fragmentation and abrasion); (2) daughter fragments derived by fragmentation of parent particles wherein the daughter fragments increase in abundance and are reduced in median grain size with increased residence time^18^; and (3) ultra-fine (<4 μm) chips produced by abrasion of irregular particle morphologies. These ultra-fine chips keep a constant median diameter of ~2.5 μm, but increase in abundance with longer jet residence times.

### TGSD Models

We describe the TGSD’s using a linear combination of normalized log-normal distributions^35^ (Gaussian distributions applied to the natural logarithm of particle size, *d*) representing three modes corresponding to: (1) the feed; (2) the daughter particles and (3) the ultra-fines (see Methods). The three normalized log normal distributions are fitted to the TGSD’s (Fig. 2a) as:

1 $${V}_{n}=\sum _{i=1}^{3}\frac{{p}_{i}}{\sqrt{2\pi }\,{\sigma }_{i}}{e}^{(-0.5{[ln\frac{d}{{x}_{i}}/{\sigma }_{i}]}^{2})}$$

where *V*_*n*_ is the volume particle distribution. *x*_*i*_, and *σ*_*i*_ are adjustable parameters for the 3 model distributions and represent the median particle size and standard deviation of each distribution, respectively. The third adjustable parameter, *p*_*i*_, is the weighting or proportion of each subpopulation and the sum of *p*_*i*_’s is required to equal 1. Note that here, and throughout this study, we use particle diameter, *d*, and fit *ln*(*d*) as our independent variable rather than phi [−log_2_(*d*)]. Illustrative results for a short term (1 h) and long term (16 h) attrition experiment are presented in Fig. 2b and c respectively.

The attrition experiments provide several critical insights (Fig. 2d). First, the process is efficient, such that only extremely short residence times are required to start producing abundant fines. Even within a low energy jet (10 L min^−1^), the original feed is reduced by ~50 vol. % within the first 15 minutes of attrition. Within 1 hour the original feed is reduced to ~25 vol.% by conversion to secondary daughter fragments (~62%) and ultra-fines (10%). Second, the decay of the parental mode (feed) and growth of the two secondary peaks are proportional to time. With increased time and attrition, the feed volume diminishes as it creates daughter particles by fragmentation whilst continual abrasion of all particle surfaces entrained into the jet continually produces ultra-fine chips. All experiments were conducted at room temperature. However, we do not expect attrition productivity to be substantially different at magmatic temperatures. Within volcanic conduits and plumes the timescales of collisional interactions between ash particles are short relative to the melt’s relaxation timescale, even at temperatures above glass transition temperatures (i.e. magmatic). The consequence is that the particles behave as brittle solids^26^.

### Surface Area Analysis

**Figure 3:**
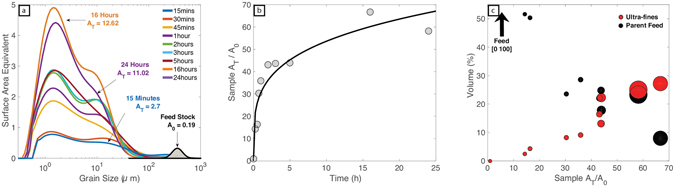
Surface area implications of particle size distributions shown in Fig. 2. (**a**) Surface area distributions calculated from grain size volume % datasets (see text) as a function of particle size for both the parent feed stock (grey shaded field) and experimental run products (solid curves). Surface area distribution curves colour-coded for duration (15 min to 24 h) of attrition experiments; A_T_ denotes the area under each curve. (**b**) Calculated total surface area of each experiment (A_T_) normalised to surface area of the original pumice particles (A_0_) and plotted against experimental attrition time. A_T_/A_0_ increases rapidly over the first 3–4 hours before reaching a plateau value. (**c**) Calculated values of A_T_/A_0_ plotted against the volume % (cf. Fig. 2d) of parent feed (black) and ultra-fines (red). The size of symbols denotes the attrition duration; larger circles indicate a longer experiment duration.

Attrition, especially abrasion produced surface chips, can substantially increase the particle surface area exposed to the gas-pyroclast suspension within the conduit and eruption plume. This increase in surface area attending attrition-driven ash production allows for more efficient heat transfer^36^, by increasing surface area to volume ratio, and thereby cooling of the plume but, more importantly, increases the overall chemical reactivity of the eruption column^37–39^. We have modelled the evolution and increase in total surface area during the attrition experiments. We converted experimental TGSD curves (Fig. 2a) to an equivalent surface area by assuming spherical particles (Fig. 3a). The model values of total surface area (A_T_) reported in Table 1 are calculated from the area under the surface area-grain size curves (Fig. 3a). They provide a quantitative estimate of surface area increase which we have portrayed as values of A_T_ normalised to the surface area calculated for the original feed (A_0_; Table 1; Fig. 3b). Surface area production in the attrition experiments is efficient and causes total surface area to increase by two orders of magnitude. Furthermore, surface area generation is fast; the surface area of the initial feed (A_0_) is increased to 14A_0_ within 15 min and to 66 times greater than the feed during 16 hours of attrition (Fig. 3b). As milling proceeds the volume of original feed particles decreases whilst the volume of ultra-fines increases (cf. Fig. 2d). This production of ultra-fine particles with a high surface area to volume ratio is responsible for elevating the bulk surface area of the experimental tephra products (Fig. 3c).

**Table 1:** Surface area data from the attrition experiments. A_T_ is the area under the surface area equivalent vs. grain size curves in Fig. 3a and A_0_ is the surface area of the initial parent feed.

Duration (h)	Surface Area	
	A_T_	A_T_/A_0_
**0**	0.189	1.00
**0.25**	2.70	14.28
**0.5**	3.08	16.29
**0.75**	5.74	30.35
**1**	6.78	35.85
**2**	8.15	43.10
**3**	8.27	43.73
**5**	8.30	43.89
**16**	12.62	66.74
**24**	11.02	58.17

### Power-law Analysis

**Figure 4:**
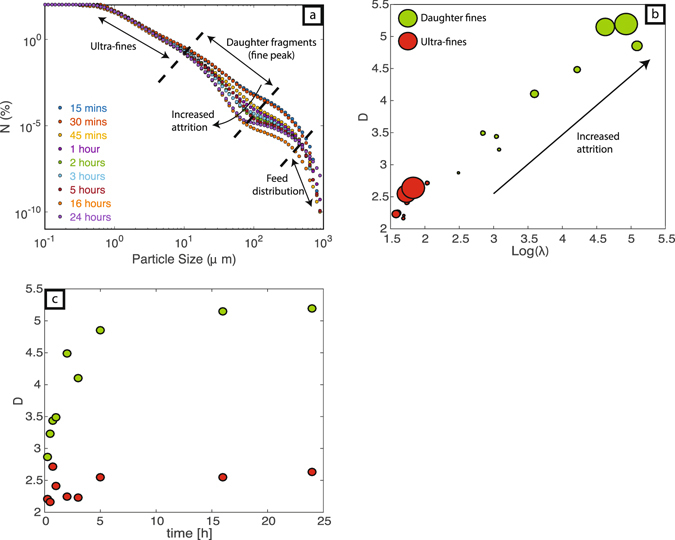
(**a**) Cumulative particle abundance as a function of grain size and fitted to the log linear relationship log N = −D*log size +λ. (**b**) Model values of D vs. λ; symbol size is proportional to experimental time. (**c**) Model values of D as a function of attrition duration (h) of attrition experiments for the daughter (green) and ultra-fine (red) particles.

Power-law representations (Fig. 4) provide a means to compare results from the attrition experiments. Plotted as particle number density *vs*. size the three grain size sub-populations (i.e. parent feed, daughter fragments and ultra-fines) define two breaks in slope, strongly suggesting three different mechanisms of grain size reduction^40^ (Fig. 4a). Daughter and ultra-fine particle subpopulations both exhibit fractal behaviour characterized by unique slopes (D) and intercepts (λ) (Fig. 4b). The daughter particle fragments display a range of D and λ values; with increasing attrition both D and λ increase to maximum values of 5.2 and log (5) respectively. Corresponding values for the ultra-fine sub-population, however, remain near constant with increased attrition (D ~2.4 and λ ~1.7).

Previous experimental work has shown that fractal dimensions can increase by secondary fragmentation during transport in PDC’s or by collisions within the conduit^11, 12, 14^. Here, we show that increased attrition within a particle loaded gas suspension causes a dramatic increase in both D and λ for the daughter fragment population. Daughter fractal dimensions are increased to values >4 in residence times >2 h. This suggests size modification processes are most effective at early residence times. In contrast, over the same range of residence times, values of D and λ for the ultra-fine population remain near constant (Fig. 4c; ~2.4). We suggest these near-constant D values result from increasing production of surface chips having a uniform size that is independent of residence time. Once formed by abrasive processes, the ultra-fine particles (i.e. surface chips) are removed to the isolated collection box without further size modification and only increase in abundance.

## Discussion

Fundamentally, particle attrition is controlled by the frequency or rate of collisions and their magnitudes For any system, natural or experimental, attrition is therefore controlled by a minimum of four governing properties: the particle concentration suspended in the jet; the feed particle size distribution; the particle/gas velocity; and the particle residence time. Attrition is promoted by high particle concentrations, poorly sorted feed distributions, high differential velocities, and long residences times. Our analysis of the grain size data from pumice attrition experiments has shown grain size reduction and ultra-fine production to occur on short timescales. In all experiments, attrition is highly efficient despite the restricted experimental conditions (mass flux input to jet of ~6 × 10^−5^ kgs^−1^ and gas velocity of 0.173 ms^−1^)^18^ which are substantially less energetic than those found in natural eruptions (mass flux rates of 10^3^–10^8^ kgs^−1^ and gas velocity of 200–500 ms^−1^)^6, 41^ fully supporting attrition within the volcanic conduit.

However, the existence of particle attrition within the eruption plume remains an open research question. During the 1980 Mt. St. Helen’s eruption, plume rise velocities of 28 ms^−1^ (the initial rising eruption column^42^) were reported. In contrast, our experiments have a superficial gas velocity of 0.173 ms^−1^; two orders of magnitude smaller than the natural case. Radar studies^43^ of the 1980 Mt. St. Helen’s eruption measured the 6-hour mean ash concentrations in the eruption plume to be 3.6–4.9 gm^−3^, whereas in our experiments bulk ash concentration is calculated as ~4000 gm^−3^, three orders of magnitude larger than the natural case. Although particle concentrations are much lower in the natural case, the gas velocities are much greater than those in our experiments. However, the particle residence times in our experiments associated with substantial attrition (≤ 2 h) are comparable to particle residence times in the volcanic plume (based on fallout times^43^). We suggest that the trade-off between higher velocities and lower particle concentrations in volcanic plumes combined with substantial residence times will support ash production by attrition. Clearly, attrition will be maximized in volcanic plumes where particle concentrations are highest and gas velocities are highest (e.g. the vent proximal gas thrust region). On this basis, our experiments and analysis strongly support previous models for *“plume and conduit attrition”* attending eruptions^11, 12, 14, 26, 27, 44^ and we suggest that it is a process that operates within all explosive volcanic eruptions. We expect attrition to be most successful within the conduit and decrease in efficiency within the eruption plume as particle concentrations are reduced and vulnerable, highly irregular, particle exteriors are removed.

**Figure 5:**
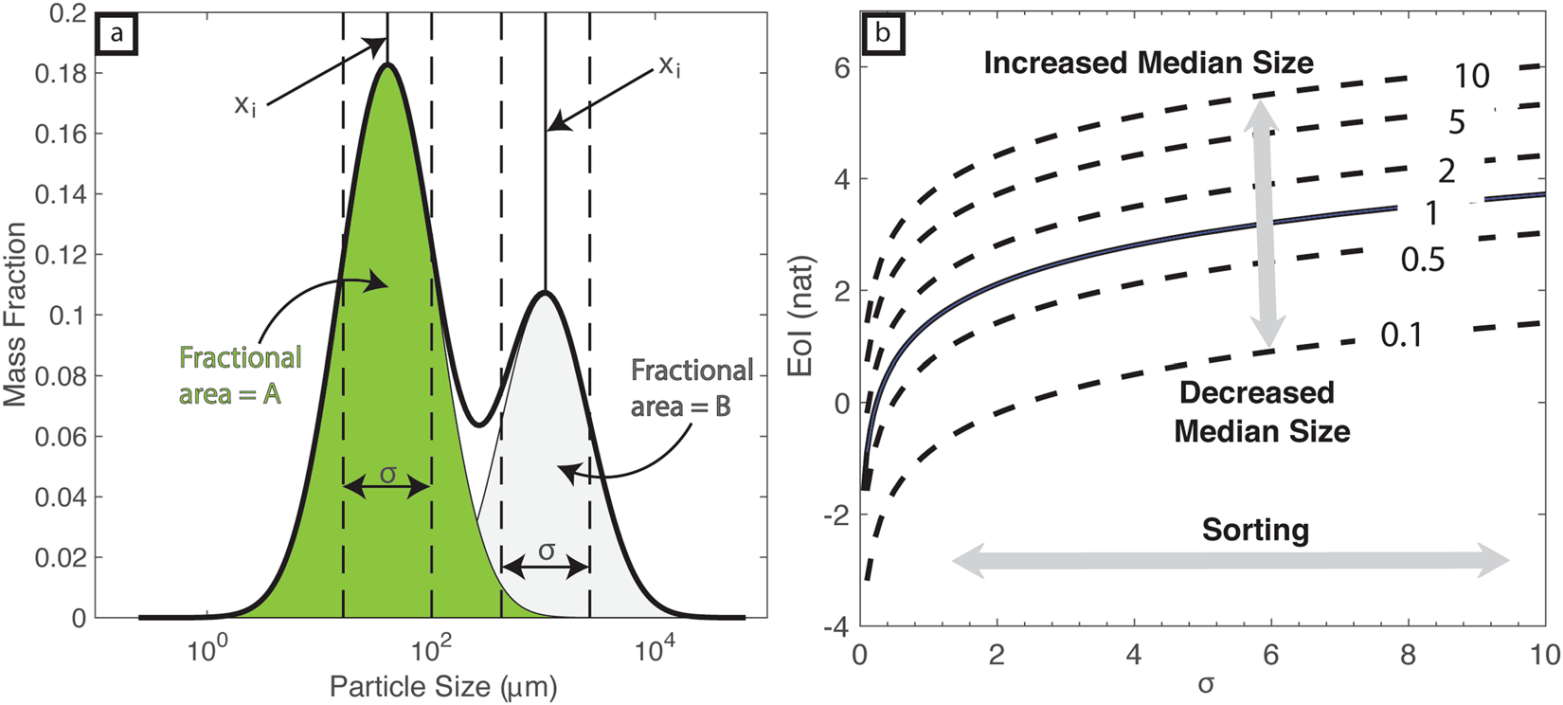
Entropy of information (EoI) calculation method for experimental and natural grain size distributions. (**a**) Log normal grain size distribution for a natural sample labelled for parameters used to compute values of EoI. The weighted EoI of the bimodal distribution is calculated as: $EoI=A[\frac{1}{2}+\frac{1}{2}\,\mathrm{ln}(2\pi {\sigma }_{i}^{2})+\,\mathrm{ln}({x}_{i})]\,+B[\frac{1}{2}+\frac{1}{2}\,\mathrm{ln}(2\pi {\sigma }_{i}^{2})+\,\mathrm{ln}({x}_{i})]$. Where A and B are the fractional areas summing to 1. (**b**) Computed EoI values for a range of standard deviations (degree of sorting). Arrows indicate how changing the median grain size and degree of sorting independently change the EoI value. The median grain size contours are in the same user defined units as $\sigma$ (e.g., µm, mm, cm).

To compare our experimental insights to natural eruptions we now introduce a metric that facilitates comparison of TGSDs (i.e. between natural deposits or *versus* experimental data). The time dependent changes in the experimental TGSD’s (Fig. 2) can be fully described by the median particle sizes (*x*_*i*_) and standard distributions (*σ*_*i*_) of a series of log normal distributions (Fig. 5a). One way to classify these distributions and quantify how they vary through time is to consider the disorder they contain. To do this we use the Entropy of Information (EoI), which can be approximated on a log-normal size distribution function as:

2 $$EoI={\int }_{0}^{\infty }f(d)\mathrm{ln}(\frac{1}{f(d)})\approx 0.5+0.5ln(2\pi {\sigma }_{i}^{2})+ln\,{x}_{i}$$

where *f*(*d*) is the normalized mass density function of particles. It is related to the Shannon entropy but has been expanded in this study for probability density functions^45^. The entropy of information has the SI units of nats (the natural unit of information). EoI serves as a measure of the disorder associated with a random variable; in this case, particle size is the bounded random variable^45^. Figure 5a shows how we used the information stored in a TGSD for both natural and experimental samples to compute the EoI. When considering input terms (*x*_*i*_, *σ*_*i*_) independently, better sorting, quantified by smaller standard deviations reduces the EoI; however, this effect is non-linear and is much more pronounced at low *σ*_*i*_ values (Fig. 5b). Also, increasing *x*_*i*_ acts to increase the EoI. In general, high values of EoI represent particle populations that have large (relative) median diameters and are poorly sorted (Fig. 5b). To a certain extent, more disorder in particle size means a greater potential for more breakage-inducing collisions and thus a higher rate of attrition. Conversely, deposits having low values of EoI represent a more-refined (well sorted) particle population of finer grain size and, thus, less potential for further milling^45^. On this basis, the EoI can be thought as a metric to quantify the residual milling potential of a particle population.

**Figure 6:**
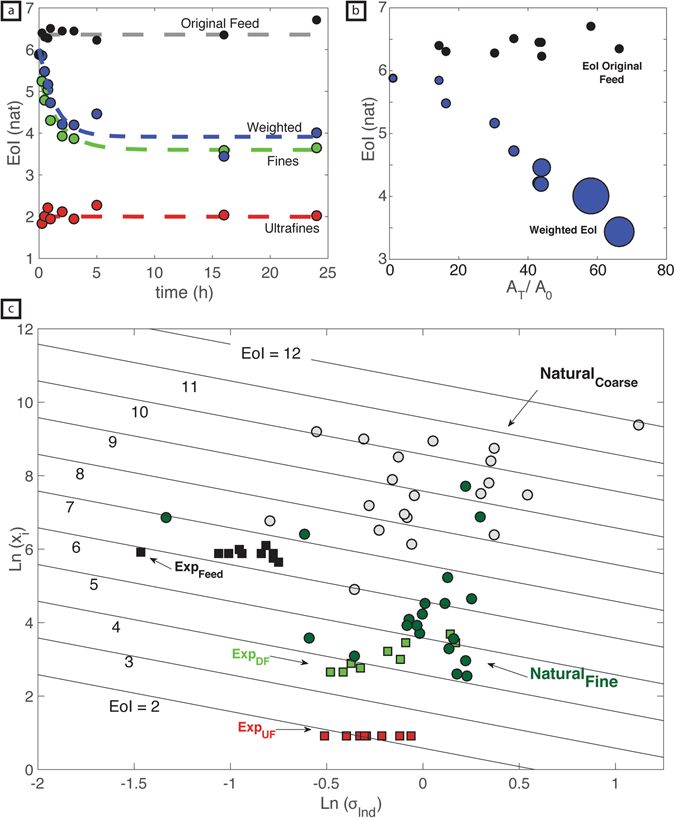
(**a**) Values of EoI calculated for the three log normal distributions fitted to each attrition experiment and plotted against time (h), including: i) the modified original feed, ii) the daughter particles, and iii) the ultra-fines (Data available in Table 2). The attritted feed reduces in abundance but maintains a constant EoI ~6.36. EoI for the daughter particles decreases with time to a fixed value of 3.6. The ultra-fines have a constant EoI (~2.04). The weighted aggregate value of EoI decreases to ~4. (**b**) Calculated values of A_T_/A_0_ plotted against the EoI of parent feed (black) and the weighted sample (blue). The size of symbols denotes the milling duration; larger circles denote longer durations. (**c**) Natural logarithms of median particle size and standard deviation from the model log normal distributions fitted to experimental (Table 2) and natural data (Fig. 1; Table S1); diagram is contoured for values of EoI (2–12). Grain size distributions for each experiment are modelled as a combination of 3 log normal distributions; natural data (Fig. 1) are modelled as 2 log normal distributions (Fig. 5a).

Here, we firstly describe EoI values calculated from the well-controlled attrition experiments which provides a basis for discussing the EoI of natural volcanic tephra. Within the experiments, the weighted EoI (blue line; Fig. 6a) decreases with time resulting from two factors: (1) the production of daughter particles (~40 µm) by fragmentation and lowering of their median particle size by subsequent, continued abrasion. Specifically, *x*_*i*_ of the daughter fragments reduces from 39.91 µm at 15 mins of attrition to 14.16 µm after 24 hours of attrition (Table 2). Furthermore, continued abrasion of these products also reduces the distribution’s variance producing a stable EoI of ~3.6 for this subpopulation (green line; Fig. 6a). (2) Continued abrasion of all particles entrained into the jet creates an ultra-fine subpopulation (EoI ~2; red line; Fig. 6a). We note that the EoI value for the ultra-fine population is independent of attrition time. This is because under ASTM experimental conditions^18^ 2.5 µm is the stable median chip size produced; furthermore σ_*i*_ remains constant due to hydraulic sorting during transport to the isolated fines collection bin (Table 2). It is the growth in abundance of the ultra-fine population and reduction of the parental feed with increased attrition, that reduces the bulk or weighted EoI of the TGSD to ~4. This is further supported by our surface area analysis; as the relative surface area (A_T_/A_0_) of the experimental products increases due to the production of ultra-fines the weighted EoI is observed to decrease systematically (Fig. 6b).

**Table 2:** Entropy of Information model parameters and results for all experimental samples.

Duration (h)	Distribution 1 (Ultra-Fines)				Distribution 2 (Daughter Fragments)				Distribution 2 (Parent Feed)				Weighted EoI
	Proportion	Median (µm)	S.D.	EoI	Proportion	Median (µm)	S.D.	EoI	Proportion	Median (µm)	S.D.	EoI	
**0**	0.000	—	—	—	0	—	—	—	1	375	0.231	5.88	5.88
**0.25**	0.025	2.5	0.600	1.83	0.477	39.91	1.154	5.25	0.516	317.0	0.460	6.40	5.85
**0.5**	0.043	2.5	0.720	2.01	0.464	31.70	0.914	4.79	0.504	282.5	0.472	6.31	5.48
**0.75**	0.082	2.5	0.886	2.21	0.696	31.70	1.185	5.04	0.235	355.7	0.365	6.28	5.17
**1**	0.091	2.5	0.673	1.94	0.624	20.00	0.889	4.30	0.286	355.7	0.459	6.51	4.72
**2**	0.164	2.5	0.808	2.12	0.619	17.83	0.689	3.93	0.221	399.1	0.385	6.45	4.21
**3**	0.131	2.5	0.672	1.94	0.608	15.89	0.723	3.86	0.248	355.7	0.431	6.45	4.20
**5**	0.223	2.5	0.939	2.27	0.637	25.18	0.832	4.46	0.178	355.7	0.346	6.23	4.46
**16**	0.272	2.5	0.746	2.04	0.661	14.16	0.619	3.59	0.080	355.7	0.390	6.35	3.44
**24**	0.250	2.5	0.738	2.03	0.531	14.16	0.660	3.65	0.233	447.7	0.442	6.71	4.01

We have applied the Entropy of Information concept to TGSDs recalculated^6^ for deposits of volcanic tephra derived from a wide range of eruption styles, magma viscosities, plume heights and mass eruption rates. Figure 1 illustrates a selection of these^6^ and the full dataset is available in Table S1. The modelled EoI for all natural eruptions show high values for the coarse fraction and low values for the finer subpopulation (Fig. 6c). The experimental data demonstrate that the median fragment size (*x*_*i*_) informs on the generative fragmentation mechanism/energy whereas the population variance (σ) informs on the degree of attrition (mainly abrasion). During a volcanic eruption, primary fragmentation creates a fragment size distribution with high variance (σ) but characterized by a coarse median grain size, *x*_*i*_. This population of particles is analogous to the experimental feed; with high σ values it is capable of undergoing substantial attrition. The initial *x*_*i*_ reflects the energy of primary fragmentation, with higher energies creating finer GSDs^8, 9^ (Fig. 6c). Then, secondary fragmentation of pyroclasts operating above the fragmentation surface creates a sub-population of daughter particles with a lower EoI. From our experimental results we suggest that, with increased transport time and distance, the fractional volume of daughter products contributing to the TGSD increases. By simple mass balance arguments, the increase in daughter products (low EoI) acts to lower the total system entropy. The EoI, therefore, can quantify the degree of attrition represented by individual GSD’s and for a single volcanic system, lower EoI can be taken as evidence of, and resulting from, greater particle attrition during transport.

Primary magmatic fragmentation produces particles that are variably susceptible to secondary fragmentation. The tephra is immediately entrained into a particle-laden high-velocity rising jet comprising an ideal environment for the efficient milling of particles^12, 26, 27^. The production of fines, by attrition, starts in the subterranean conduit^11, 12, 14^ and potentially extends to within the volcanic plume. We suggest this environment easily transforms a coarse primary particle size distribution (with low D, high EoI) to one which is heavily enriched in fine ash (high D, low EoI)^11, 14^. In detail, disruptive collisions^12^ between parent particles lead to fragmentation, creating a new subpopulation of particles with a finer median grain size. Continued entrainment and transport within the conduit and plume supports continued lower energy collisions^12^ and abrasion of irregular particle exteriors^15^. Fine ash production is most successful when primary fragmentation is deep, particle density is high^12, 14^, and eruption columns are high.

Our model shows that attrition and fine ash generation are inevitable in any explosive volcanic eruption. We show that longer residence times in a particle-laden jet increase fine ash production. Hence, volcanoes with deep fragmentation depths and large mass eruption rates (high plumes^46, 47^) are particularly susceptible to sustained attrition during transport. We extend and support previous work^12^ to show that attrition can result in pervasive modification of the entire grain size population produced at the fragmentation surface and therefore biases our view of TGSD’s and the associated deposits. Currently, TGSD’s form our best approximation of the initial grain size distribution at the fragmentation front^10, 48, 49^, an extremely important eruption source parameter when forecasting atmospheric ash dispersion^50, 51^. However, current models do not allow for the continuous, transient production of fines during conduit and plume transport. Furthermore, accurate forecasting of ash dispersion is an essential requirement for effective management of airspace during a volcanic eruption^52^. Failure to accurately forecast ash dispersion has implications for: ash ingestion leading to potential failure of jet engine turbines^1, 2, 53^; the production of tephra fallout hazard maps, and the spatial extent of atmospheric ash loading resulting in short-term climate change. Secondary fragmentation upon transport should be considered before interpreting TGSDs.

The sustained attrition-driven production of fines within the eruption plume has substantial implications for plume dynamical models^54^ and other processes that operate within the eruption plume and atmosphere. For instance, the generation of volcanic lightening is known to be most effective when abundant fines are present^55^. Given that volcanic lightening is rapidly gaining use as a hazard-forecasting tool it is important to truly understand the fine ash contents of plumes. Also, increased loading of fine ash into an eruption plume may lead to regions of slower flow at the vent and a greater chance of PDC formation by column collapse^12, 56^. Increased concentration of fines within the eruption plume may also enhance ash aggregation^31^, thereby increasing the effective particle size and resulting in premature fine ash fallout. The reduction of median particle size and increase in surface area can drastically increase the opportunity for HCl and SO_2_ scavenging and injection into the stratosphere^57, 58^. Furthermore, the increased surface area to volume ratio of fine ash may enhance the Fe input when deposited in ocean surface waters; an important feedback into the Earth’s biogeochemical cycle^59, 60^.

Our model has shown that ash generation by attrition processes is an inevitable consequence of many, if not all, explosive volcanic eruptions. Attrition occurs upon rapid timescales even in less energetic, less particle rich conditions to those expected with eruption plumes. We have introduced the Entropy of Information (EoI) as a valuable metric that quantifies the attrition potential or history of a grain size distribution. For a single volcanic system, TGSDs with low EoI are likely to have experienced substantial milling whereas TGSDs with high EoI values correspond to a particle population yet to be attritted. We have shown that abrasion documented by the formation of abundant surface chipping^18, 61^ drastically increases the ash surface area available for biogeochemical processes. Within plume and conduit, attrition is a highly efficient form of secondary fragmentation transforms our view of TGSDs and must be considered in aspects of dispersion and plume dynamical models for us to truly understand the volcanic fragmentation.

## Methods

### Sample preparation for Attrition Experiments

The samples used in our attrition experiments were produced by crushing blocks (>10 cm) of pumice. The pumice blocks were collected from proximal to medial pyroclastic fall deposits resulting from the 2360 BP explosive eruption of the Mount Meager volcanic complex: a calc-alkaline stratovolcano complex situated approximately 150 km north of Vancouver, Canada^62^. The blocks were coarsely crushed and then manually dry sieved using a standard stack of Tyler sieves. The grain size fraction caught in the 250 μm mesh screen was divided into 20 g aliquots and stored in an airtight container ready for the experiments.

### Jet attrition experiments

All experiments were performed at ambient room temperature and pressure in a jet attrition rig with dry air fed at 10 L min^−1^ conforming to the ASTM D5757-00 method^63, 64^. The experimental apparatus comprises a basal distributor plate with three orifices 0.397 mm in diameter on which the initial sample is loaded. A 710 mm long, 35 mm internal diameter stainless steel attrition tube is connected directly above the distributor. The top of the attrition tube is capped by a wider settling chamber (110 mm in the center) and allows for large particles to settle and return to the attrition tube. The upper cone of the attrition chamber is connected to a fines collection bin. A gas exit pipe with a ceramic filter (~0.1 μm mesh) allows gas to flow unimpeded through the fines collector bin.

For each experiment the distributor plate, gas feed and attrition tube were connected, after which a 20 g sample of 250–500 μm pumice was introduced. Specifically, the sieved pumice sample was poured down the attrition tube to rest on the distributor plate after which the settling chamber and fines collector were connected. Dry compressed air was connected via a calibrated rotameter and increased to a flow rate of 10 L min^−1^ whilst checking for leaks. We performed experiments for 0.25, 0.5, 0.75, 1, 2, 3, 5, 16 and 24 hours. At the end of each experiment, the gas was switched off and the sample material was left to settle for at least 1 hour after gently knocking the apparatus to loosen adhering fine particles.

Particles from each experiment were collected in three stages. Firstly, the fines collector was removed and the ultra-fine contents were brushed into a storage container. Secondly, the *empty* fines collector, connecting pipes, and the settling chamber were rinsed at least twice with deionised water over a 63 μm sieve to remove any remaining ultra-fines adhering to the walls of the apparatus. The distributor plate was then removed within a sample collection bag, making sure to brush all of the bolts/washers to recover the entire sample. Lastly, the attrition tube was flushed again with deionised water over a 63 μm sieve.

### Post-attrition sample characterisation

All of the recovered sample was added to deionised water and measured using a Malvern Mastersizer 2000 with the hydro 2000 Mu water dispersion module attached (capable of measuring particles 0.02 μm to 2000 μm in size.) Using a pump speed of 1900 rpm an aliquot of the attrition sample was added to the dispersion module and measured three times. An ultrasonic pulse was applied to the sample for 2 s before the measurement to prevent particles from aggregating in the water suspension. For each experimental product three separate aliquots were taken, each measured three times; therefore the results presented represent averages of nine measurements.

### Calculation of EoI Values from Model Grain Size Distributions

The Entropy of Information (EoI) is a measure of disorder associated with a random variable. The EoI concept can be applied to grain size distributions found in natural pyroclastic deposits if particle size is taken as the bounded random variable. As discussed in the main text, values of EoI can be approximated from the log-normal size distribution functions used to describe the grain size variations within individual deposits or total grain size distributions estimated for individual volcanic eruptions. Specifically, EoI values are computed from the median particle size (*x*_*i*_) and standard distribution (*σ*_*i*_) of log normal distributions fitted to the grain size data:

3 $$EoI\approx 0.5+0.5\,ln(2\pi {\sigma }_{i}^{2})+ln\,{x}_{i}$$

Higher values of EoI (more disorder) coincide with larger medians and higher variances of particle sizes reflecting a greater potential for particle size reduction and, thus, higher rates of attrition.

Jones *et al*. (2017)^18^ presented a series of analogue experiments wherein they used an air jet to mill pumice tephra under fixed conditions for variable amounts of time. Total grain size distributions are published for the run products from each experiment allowing us to compute their respective EoI values following the methods advanced by Xiao *et al*. (2014﻿)^45^.

The initial material (i.e. feed) is modelled as a unimodal grain size distribution having a median of 375 μm and 3 standard deviations equal to 250 μm (see Fig. 2a). The EoI for the feed is 5.88. Subsequently, the total grain size distributions (vol. % *vs*. size) from each experiment were fitted to a normalized log-normal distribution comprising up to three separate distributions defined by modes in the datasets expressed as vol. % *vs*. *ln* diameter (Fig. 2c and d). We assume that the modes define the median particle size (*x*_*i*_) for each distribution, that each log normal distribution is symmetric about the mode, and that the proportions (*p*_*i*_) of each of the distributions sum to 1. For a model comprising 3 normal distributions, the adjustable parameters are the values of *σ*_*i*_ and *p*_*i*_ (*i* = 1:3). The model values of *x*_*i*_ and *σ*_*i*_ allow us to compute EoI values for each distribution representing the modification of the original feed, the production of daughter particles and the production of ultra-fines (Fig. 6); the model values of *p*_*i*_ allow for a weighted value of EoI representing the total grain size distribution (Fig. 6a).

We also applied the EoI concept to TGSD’s from natural pyroclastic deposits previously compiled by Costa *et al*. (2016)^6^. Costa *et al*. (2016)^6^ modelled the TGSD’s as the sums of 2 log-normal distributions (i.e. Gaussian in the parameter phi (ϕ) = −log_2_ d). We used their model values of mean, standard deviation and proportion for the 2 distributions to recalculate the distributions in natural logarithm space. We then refit the TGSD’s to a bimodal normalized log-normal distribution (based on the natural logarithms of particle size) to obtain corresponding values of *x*_*i*_, *σ*_*I*_, and *p*_*i*_. These model values were used to compute EoI values for the natural data that then could be compared to the experimental data (Fig. 6).

### Data Availability

The datasets generated during and/or analysed during the current study are available from the corresponding author upon request.

## Electronic supplementary material

Supplementary Table S1
